# Reducing Infections and Improving Healing in Complex Wounds: A Systematic Review and Meta-Analysis

**DOI:** 10.3390/jcm14093237

**Published:** 2025-05-07

**Authors:** María Juana Millán-Reyes, Diego Fernando Afanador-Restrepo, María del Carmen Carcelén-Fraile, Agustín Aibar-Almazán, Marcelina Sánchez-Alcalá, Javier Cano-Sánchez, María Aurora Mesas-Aróstegui, Yolanda Castellote-Caballero

**Affiliations:** 1 University Hospital of Jaén, 23007 Jaen, Spain; mjmr0013@red.ujaen.es; 2 Department of Health Sciences, Faculty of Health Sciences, University of Jaén, 23071 Jaen, Spain; 3 Faculty of Health Sciences and Sport, University Foundation of the Área Andina—Pereira, Pereira 660004, Colombia; 4 Faculty of Distance and Virtual Education, Antonio José Camacho University Institution, Santiago de Cali 760016, Colombia; 5 Department of Education Sciences, Faculty of Social Sciences, University of Atlántico Medio, 35017 Las Palmas de Gran Canaria, Spain; 6 Pediatric Endocrinology Department, Instituto Hispalense de Pediatría, Hospital Quirón Marbella, 29603 Malaga, Spain; 7 Pediatrics Department, Hospital of Guadix, 18500 Granada, Spain; 8 Department of Health Sciences, Faculty of Health Sciences, University of Atlántico Medio, 35017 Las Palmas de Gran Canaria, Spain

**Keywords:** chronic wounds, traumatic injuries, wound healing, surgical site infections

## Abstract

**Background and Objectives**: Wound management in complex and traumatic injuries remains a significant clinical challenge, with a high risk of surgical site infections (SSIs) and associated complications. This systematic review and meta-analysis aim to evaluate the effectiveness of diverse interventions, including Negative Pressure Wound Therapy (NPWT), advanced dressings, irrigation techniques, antibiotic regimens, and innovative therapies, in improving wound healing outcomes and reducing infection rates. **Materials and Methods**: An exhaustive literature search focused on the use of NPWT, dressings, and antibiotics in the care of chronic wounds was conducted following PRISMA guidelines in the PubMed, Scopus, Web of Science, and CINAHL databases. Eligible studies included randomized controlled trials and prospective cohorts assessing interventions for wound care in trauma, surgical, and chronic wound settings. The risk of bias was assessed using the ROB2 tool. Subgroup analyses were performed to evaluate the relative risk (RR) of infections based on the intervention type. **Results**: The analysis included 17 studies spanning diverse geographical and clinical settings. NPWT demonstrated significant benefits in reducing infection rates compared to control groups (RR: 0.590, 95% CI: 0.458–0.760, and *p* < 0.001). Although advanced dressings demonstrated clinically relevant benefits as reported across the included studies, the meta-analysis did not reveal statistically significant differences (RR: 0.516, 95% CI: 0.242–1.100, and *p* = 0.087). Antibiotic therapies significantly reduced infections when administered within 24 h of injury, while low-pressure irrigation techniques effectively minimized bacterial contamination without causing tissue damage. Growth factors and honey-based treatments exhibited promising results in accelerating wound healing and reducing infection risks in chronic wounds. **Conclusions**: NPWT emerges as a highly effective intervention for infection prevention and wound healing, supported by robust evidence. Advanced dressings and innovative therapies show potential but require further research for conclusive evidence. These findings underscore the importance of tailoring wound care strategies to the clinical context and patient needs. Future research should focus on long-term outcomes and cost-effectiveness analyses to enhance the integration of these therapies into clinical practice.

## 1. Introduction

The effective management of traumatic, surgical, and chronic wounds remains a significant challenge in clinical practice due to the high rates of infection and complications that can negatively impact patient outcomes and healthcare costs [1]. Wound care, whether addressing open fractures, surgical site infections (SSIs), or injuries from trauma, requires comprehensive therapeutic approaches that incorporate advanced technologies, meticulous surgical techniques, and innovative strategies to ensure optimal healing and minimize complications.

Over the past two decades, various interventions have been developed and evaluated to address these challenges. Among these, Negative Pressure Wound Therapy (NPWT), advanced wound dressings, irrigation protocols, and antimicrobial therapies have emerged as key tools in wound management. NPWT, also known as vacuum-assisted wound closure, has revolutionized the treatment of complex wounds by promoting granulation tissue formation, reducing edema, and decreasing bacterial load through the application of controlled subatmospheric pressure to the wound bed [2]. Studies have consistently demonstrated its efficacy in managing traumatic wounds and post-surgical healing, leading to faster recovery and reduced infection rates [3,4].

Advanced wound dressings, including silver-infused materials, hydrocolloids, and polyurethane foams, have also played a crucial role in improving wound healing outcomes [5,6,7]. These dressings maintain an optimal moist environment, manage exudates, and provide antimicrobial properties that reduce the risk of infection and promote tissue regeneration [8]. These interventions are particularly beneficial in managing wounds associated with open fractures, SSIs, and chronic conditions such as diabetic foot ulcers and pressure ulcers [9].

Additionally, early and effective irrigation protocols have been shown to significantly reduce bacterial contamination in open wounds. Low-pressure saline irrigation, for example, has been found to be as effective as high-pressure methods while minimizing tissue damage [10]. Antibiotic therapies, both systemic and topical, have further complemented these strategies by addressing infections and preventing bacterial colonization in high-risk wounds [11].

Despite the advancements in wound care, challenges remain in comparing traditional and modern approaches, evaluating cost-effectiveness, and tailoring interventions to diverse patient populations and clinical contexts [12]. Recent studies have underscored the importance of innovative therapies like NPWT in vascular surgeries and advanced topical treatments in chronic wounds [13,14]. Older research has provided foundational insights into irrigation techniques and delayed primary closure in contaminated wounds [15].

This systematic review aims to synthesize existing evidence on the effectiveness of various interventions in wound healing, focusing on infection reduction, clinical outcomes, and economic viability. By exploring studies that range from advanced surgical techniques and antimicrobial dressings to innovative therapies such as growth factors and honey-based treatments, this review seeks to provide a comprehensive perspective that supports evidence-based clinical decision-making and optimizes the care of patients with complex wounds.

## 2. Materials and Methods

This systematic review aimed to classify and analyze interventions for the treatment and management of various types of wounds, focusing on open fractures, surgical site infections (SSIs), chronic wounds, burn wounds, and traumatic injuries. The review followed the PRISMA 2020 statement guidelines and adhered to the Cochrane Handbook for Systematic Reviews of Interventions, and the protocol was registered in PROSPERO (CRD42024616047).

### 2.1. Inclusion Criteria

The studies included in this review met the following criteria: (i) randomized controlled trials (RCTs); (ii) studies investigating wound management in trauma, surgery, or chronic care settings; (iii) interventions aimed at improving wound healing and reducing complications, such as infection or delayed closure.

### 2.2. Exclusion Criteria

Studies were excluded if they did not focus on the predefined wound types pertinent to this review. Additionally, studies lacking sufficient methodological rigor or those without wound-related outcome measures were omitted. Non-primary research articles, including protocols, meta-analyses, reviews, book chapters, and non-peer-reviewed articles, were excluded to avoid the incorporation of potentially low-quality or biased information. Furthermore, studies addressing conditions unrelated to trauma or involving patient populations outside the scope of this review were not considered.

### 2.3. Information Sources

A literature search was conducted between August and October 2024 in the PubMed, Scopus, Web of Science, and CINAHL databases.

### 2.4. Search Strategy

(“Negative-Pressure Wound Therapy” OR “Suction” OR “Vacuum” OR “negative pressure” OR “negative-pressure” OR “TNP” OR “NWPT” OR “NPWT” OR “soap” OR “Irrigation” OR “Antibiotics” OR “dressings” OR “current”) AND (“Surgical Wound” OR “Surgical Wound Dehiscence” OR “Wound” OR “open fracture” OR “open fracture patient”).

### 2.5. Study Selection Process

The search results were processed using the Rayyan QCRI application [16] (https://rayyan.qcri.org/welcome (accessed on 13 March 2025)), where duplicates were automatically removed. Two authors (D.F.A.-R. and M.J.M.-R.) independently and blindly reviewed the titles and abstracts to verify compliance with the inclusion criteria. Full-text articles were then reviewed by the same authors. Any discrepancies arising during this process were resolved by consensus with a third author (J.C.-S.).

### 2.6. Data Extraction

The data extraction process focused on collecting comprehensive details from each included study to enable a thorough analysis. Key information extracted included the year of publication, the country where the study was conducted, and the author(s). Participant demographics were also collected, such as the age, the sample size, and the distribution of participants between the experimental and control groups. Specific intervention protocols were documented, including details on the type of intervention (e.g., advanced dressings, Negative Pressure Wound Therapy, irrigation techniques, or antibiotic regimens) and the types of wounds treated, such as open fractures, surgical site infections, chronic wounds, burns, or traumatic injuries. The primary and secondary outcome variables were categorized, focusing on wound healing progression, infection rates, and the occurrence of secondary complications. Finally, the duration of follow-up and the metrics used for assessing outcomes were recorded. This structured approach allowed for a detailed comparison of interventions and their effectiveness across diverse patient populations and wound types.

### 2.7. Risk of Bias

The risk of bias was assessed using the Risk of Bias 2 (ROB2) tool, which was developed by the Cochrane Collaboration. The methodological quality of the randomized controlled trials was evaluated by examining five key domains: the randomization process, deviations from intended interventions, missing outcome data, the measurement of outcomes, and the selection of reported results. Each domain was assessed using specific signaling questions, and studies were categorized as having a “low risk”, “some concerns”, or a “high risk” of bias.

### 2.8. Decisions for the Meta-Analysis

The findings of the meta-analysis are displayed in a forest plot, which includes details such as the lead author, publication year, individual risk ratios (RRs), and the overall RR along with its 95% confidence interval and corresponding *p*-value. The decision to use a random effects model was based on the heterogeneity and variability determined through Cochrane’s Q test and the I^2^ statistic.

For stratified or subgroup analyses, studies were categorized according to the type of interventions used. Separate meta-analyses were then conducted for each group. The risk of publication bias was evaluated through a funnel plot.

## 3. Results

**Figure 1:**
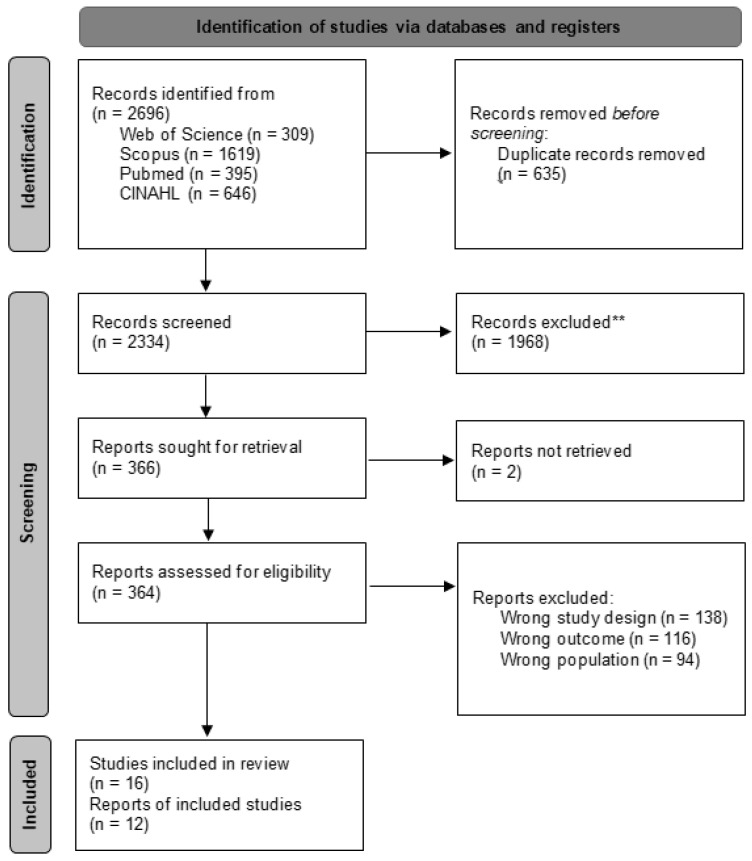
Flow diagram of the study selection process. ** studies that did not meet the basic inclusion criteria established for this review.

The initial search across various databases yielded a total of 2969 articles. After removing duplicates, 635 unique articles remained. These were subjected to a title and abstract screening, resulting in 366 articles selected for a full-text review, of which 16 articles [17,18,19,20,21,22,23,24,25,26,27,28,29,30,31,32] were ultimately included in this systematic review. The selection process of the articles, following the PRISMA guidelines, is illustrated in Figure 1.

### 3.1. Risk of Bias

Recent studies, such as those by Stanirowski et al. [24], Svensson-Björk et al. [31], Svensson-Björk et al. [32], and Stannard et al. [27], exhibit robust methodologies with a low risk of bias in domains like random sequence generation, allocation concealment, outcome data handling, and outcome measurement and reporting. These studies provide reliable evidence that strengthens the validity of their findings.

Studies like Blackham et al. [19], Rezzadeh et al. [23], and Virani et al. [30] present some concerns in specific areas such as deviations from intended interventions, the measurement of outcomes, or the selection of reported results. While these studies are methodologically sound in most domains, addressing these concerns in future research could improve the reliability of their findings.

In contrast, some older studies, including Anglen et al. [17] and Lawrentschuk et al. [21], demonstrate a moderate to high risk of bias in domains such as random sequence generation and allocation concealment. These limitations may affect the interpretability of their findings and should be considered when comparing their results with those of more recent research.

Additionally, studies such as Arti et al. [18], Blum et al. [20], Ondari et al. [22], Stannard et al. [25], Stannard et al. [26], Tauber et al. [28], and Vargo et al. [29] display a mix of low and moderate risk of bias across domains, particularly in areas like randomization and reporting. These studies, while contributing valuable insights, could benefit from enhanced methodological transparency in future investigations (Table 1).

### 3.2. Study Characteristics

All the studies included in this systematic review were conducted across various countries and clinical settings, employing diverse methodologies such as randomized controlled trials, prospective cohort studies, and cost-effectiveness analyses. The studies were published in locations such as the United States [17,25,26,27], Sweden [31,32], Australia [21], Iran [18], and Kenya [22]. Despite the geographical diversity, the studies shared a focus on managing wounds associated with open fractures, surgical site infections (SSIs), and traumatic injuries through innovative therapies like Negative Pressure Wound Therapy (NPWT), advanced dressings, and antibiotic regimens.

The studies spanned from 2002 to 2022, with notable clusters of publications between 2012 and 2016. Lawrentschuk et al. [21] examined wound blister management post-hip surgery. In 2005, Anglen [17] explored irrigation techniques for open fractures. By 2006 and 2009, Stannard et al. [26] published critical research on NPWT in severe trauma and open fracture management. The subsequent years saw increased activity, with contributions such as Blum [20] evaluating advanced dressings, Tauber [28] emphasizing meticulous surgical techniques, and Rezzadeh et al. [23] exploring local growth factors for wound healing. More recently, Svensson-Björk et al. [31] assessed the cost-effectiveness of NPWT in vascular surgeries, while Svensson-Björk et al. [32] evaluated its impact on preventing SSIs following EVAR procedures (Table 2).

### 3.3. Study Characteristics

#### Intervention

The studies included in this systematic review employed a variety of interventions aimed at improving wound management and reducing the risk of surgical site infections and complications in traumatic injuries. Each study compared an experimental intervention with a control group, such as standard dressings or no specific intervention, to evaluate its effectiveness.

Advanced dressings were a central focus across multiple studies. Blum et al. [20] highlighted the use of polyurethane foam dressings, demonstrating their effectiveness in minimizing exudate and promoting tissue granulation in trauma wounds. Blackham et al. [19] expanded on this by combining antimicrobial agents with advanced dressings, showing improved bacterial control and better healing outcomes. These dressings were typically applied immediately after injury or surgery and were changed at regular intervals, depending on the study. Virani et al. [30] investigated multilayer and silver-based dressings in managing open fractures, reporting significant reductions in infection rates and enhanced wound healing times.

NPTW was identified as a cornerstone intervention for complex wound management. Stannard et al. [25] and Stannard et al. [26] reported significant reductions in wound size, fewer hospital readmissions, and lower infection rates when NPWT was compared to standard dressings in trauma cases. Svensson-Björk et al. [32] and Svensson-Björk et al. [31] extended the application to vascular surgeries, demonstrating cost-effectiveness and reductions in infections. These studies consistently supported the use of NPWT as an effective intervention across diverse clinical settings.

Irrigation techniques were another frequently studied intervention. Anglen et al. [17] and Vargo et al. [29] compared low-pressure saline irrigation with high-pressure methods for cleaning open fractures. Both studies concluded that low-pressure irrigation effectively reduced bacterial load while minimizing tissue damage. Arti et al. [18] further examined the timing of irrigation, emphasizing the importance of early wound irrigation within the first six hours post-injury to significantly reduce infection rates.

Lawrentschuk et al. [21] compared immediate versus delayed primary closure methods. Delayed closure was associated with lower infection rates in contaminated wounds. Tauber et al. [28] emphasized meticulous tissue handling and advanced surgical planning as critical factors in minimizing surgical site infections.

Innovative approaches, such as the use of local growth factors and honey-based treatments, were explored by Rezzadeh et al. [23] and Ondari et al. [22], respectively. Rezzadeh et al. [23] demonstrated that growth factor application accelerated granulation tissue formation, while Ondari et al. [22] reported significant antimicrobial activity and improved healing with honey-based treatments.

### 3.4. Meta-Analysis

**Figure 2:**
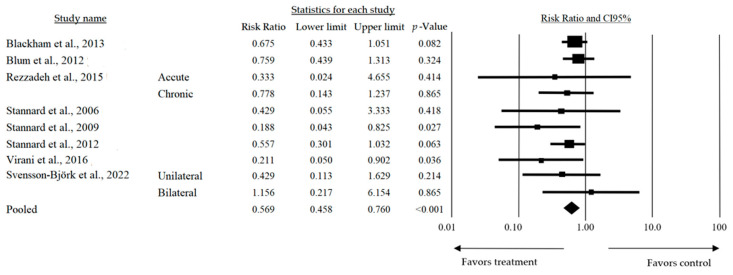
Risk ratio of NPWT use on wound infection incidence [19,20,23,25,26,27,30,32].

For this meta-analysis, a subgroup analysis was performed using the type of intervention employed—either NPWT or interventions utilizing dressings—as grouping variables. This approach allowed for the calculation of the relative risk (RR) for each subgroup. In the case of NPWT (I^2^ = 42%), eight studies were included, showing an RR that indicated the favorable effects of the intervention on reducing the number of infection cases compared to the control group (RR = 0.569, 95CI%: 0.458–0.760, and *p* < 0.001) (Figure 2).

**Figure 3:**
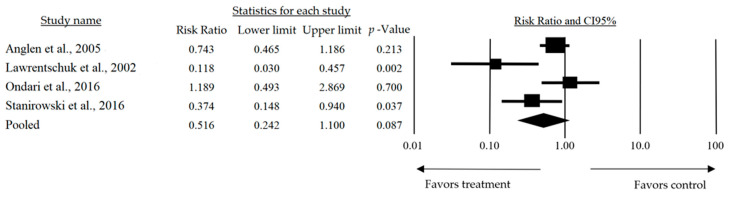
Risk ratio of the interventions utilizing dressings on wound infection incidence [17,21,22,24].

On the other hand, the subgroup analysis for interventions utilizing dressings included four studies (I^2^ = 0%), showing an RR that indicated the favorable effects of the intervention on reducing the number of infection cases compared to the control group. However, this result was not statistically significant (RR: 0.516, 95%CI: 0.242–1.100, and *p* = 0.087) (Figure 3).

## 4. Discussion

The findings of this systematic review highlight the broad spectrum of interventions studied for wound management, ranging from advanced dressings and NPTW to irrigation techniques, antibiotic regimens, surgical methods, and innovative treatments. These interventions reflect the diversity of clinical settings and patient populations included in the studies, each targeting specific challenges in wound healing, infection control, and cost-effectiveness.

Advanced dressings emerged as a pivotal intervention in wound care, demonstrating their efficacy in reducing exudate, promoting tissue granulation, and controlling bacterial contamination [33,34,35]. The incorporation of antimicrobial agents further enhanced healing outcomes, particularly in trauma-related wounds [36] and open fractures [37], where multilayer and silver-based dressings significantly reduced infection rates and accelerated wound healing [5]. Although evidence exists for other types of wounds, as previously mentioned, the results of this systematic review with a meta-analysis indicate that treatments employing dressings did not show significant differences between their control and intervention groups in the RR (RR: 0.516, 95%CI: 0.242–1.100, and *p* = 0.087) of infection occurrence in wounds.

NPWT has consistently proven to be a cornerstone intervention for managing complex wounds [38]. The studies included in this review highlighted the advantages of NPWT over standard dressings, with significant reductions in wound size, fewer hospital readmissions, and lower infection rates [25,26,27]. This effectiveness was reinforced in vascular surgeries where NPWT not only reduced infection rates but also proved cost-effective [31,32]. These findings are consistent with other systematic reviews that studied the use of NPWT in diabetic foot ulcers [39], open traumatic wounds [40], and orthoplastic surgery [41].

Irrigation techniques, particularly for open fractures, demonstrated that low-pressure saline irrigation was as effective as high-pressure methods in reducing bacterial contamination while minimizing tissue damage [17,29]. These findings align with other studies that emphasized the importance of timely irrigation within six hours post-injury to optimize outcomes [42]. Innovative treatments, such as growth factors and honey-based therapies, presented promising results [22,23]. The evidence has demonstrated accelerated granulation tissue formation with the use of local growth factors [43], which could represent a significant improvement in wound healing by reducing the risk of infection through shorter healing times. Additionally, one study [22] reported significant antimicrobial activity and enhanced healing outcomes with honey-based treatments. These findings suggest that novel therapies could complement traditional wound care methods, particularly for chronic or complex wounds [44].

The risk of bias varied among the included studies. Some of the recent investigations included [24,27,31,32] exhibited robust methodologies with a low risk of bias across key domains, providing high-quality evidence for the efficacy of interventions like NPWT and advanced dressings. However, studies like Blackham et al. [19] and Rezzadeh et al. [23] displayed moderate concerns in specific areas, such as deviations from intended interventions and outcome measurements. Older studies, including Anglen et al. [17] and Lawrentschuk et al. [21], demonstrated a higher risk of bias in randomization and allocation processes, potentially limiting the interpretability of their findings. The presence of bias in studies included in this systematic review is a significant concern that can compromise the validity of effect estimates and, consequently, influence the interpretation of pooled relative risk (RR) outcomes. Various forms of bias, including inadequate allocation concealment, a lack of blinding, and deviations from intended interventions can lead to an overestimation of treatment effects [33].

Cost-effectiveness in wound care is a crucial consideration for healthcare providers due to the increasing prevalence of chronic wounds and their associated economic burden. Al-Gharibi et al. [45] identified two key attributes of cost-effective wound care: effectiveness, such as rapid wound healing and reduced wound size, and economy, including lower treatment costs. Factors influencing cost-effectiveness encompass the wound type, care setting, dressing type, and patient characteristics, with consequences including improved patient prognosis, reduced economic burden, enhanced quality of life, and overall cost savings. In surgical wound management, NPWT has been explored for preventing surgical site infections; however, studies indicate that it is unlikely to be cost-effective compared to standard dressings. Therefore, while advanced wound care technologies offer potential benefits, their cost-effectiveness varies based on multiple factors, necessitating that healthcare providers consider both clinical efficacy and economic implications when selecting wound care interventions.

This systematic review has several strengths. It synthesizes evidence from a diverse range of interventions and patient populations, providing a comprehensive overview of wound management strategies. The inclusion of recent studies with robust methodologies enhances the reliability of the conclusions. Furthermore, the exploration of innovative therapies offers valuable insights into emerging trends in wound care. However, this review also has limitations. The variability in study designs, patient populations, and clinical settings makes direct comparisons challenging. The heterogeneity observed among the studies included in this systematic review is a critical factor that can significantly influence the interpretation of the pooled relative risk (RR) results. This variability may stem from differences in wound types, patient characteristics, clinical settings, intervention protocols, and outcome assessment methods. While a random effects model was employed to account for this heterogeneity, it is important to recognize that such an approach does not entirely mitigate the impact of underlying differences between studies. Heterogeneity may reflect variations in the methodological quality of studies, potentially influencing effect estimates and confidence in the results. Additionally, the presence of a moderate to high risk of bias in some studies underscores the need for methodological improvements in future research. Finally, the lack of long-term follow-up in many studies limits the understanding of sustained outcomes and potential late complications.

## 5. Conclusions

This review highlights the effectiveness of various interventions in improving wound healing and reducing infections, with NPWT emerging as particularly impactful. Innovative treatments offer promising avenues for future research, while antibiotic regimens and irrigation protocols remain integral to comprehensive wound care. Despite the methodological variability, the findings provide valuable guidance for optimizing clinical practices. Future studies should prioritize high-quality designs, longer follow-up periods, and tailored approaches to further enhance wound management outcomes.

## Figures and Tables

**Table 1:** Risk of bias of the included articles.

Study	Bias 1	Bias 2	Bias 3	Bias 4	Bias 5	Overall Risk of Bias
Anglen et al., 2005 [17]	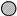 Low Risk	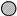 Low Risk	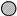 Low Risk	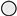 Moderate	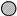 Low Risk	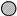 Low Risk
Arti et al., 2016 [18]	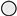 Moderate	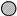 Low Risk	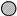 Low Risk	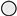 Moderate	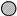 Low Risk	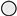 Moderate risk
Blackham et al., 2013 [19]	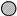 Low Risk	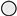 Moderate	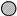 Low Risk	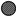 High Risk	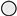 Moderate	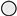 Moderate risk
Blum et al., 2012 [20]	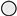 Moderate	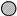 Low Risk	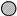 Low Risk	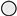 Moderate	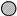 Low Risk	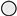 Moderate risk
Lawrentschuk et al., 2002 [21]	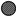 High Risk	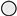 Moderate	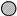 Low Risk	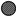 High Risk	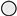 Moderate	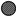 High Risk
Ondari et al., 2016 [22]	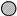 Low Risk	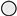 Moderate	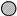 Low Risk	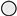 Moderate	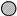 Low Risk	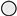 Moderate risk
Rezzadeh et al., 2015 [23]	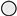 Moderate	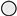 Moderate	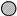 Low Risk	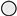 Moderate	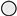 Moderate	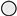 Moderate risk
Stanirowski et al., 2016 [24]	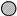 Low Risk	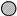 Low Risk	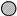 Low Risk	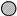 Low Risk	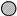 Low Risk	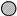 Low Risk
Stannard et al., 2006 [25]	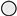 Moderate	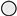 Moderate	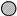 Low Risk	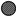 High Risk	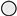 Moderate	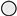 Moderate risk
Stannard et al., 2009 [26]	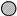 Low Risk	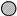 Low Risk	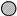 Low Risk	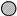 Low Risk	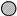 Low Risk	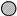 Low Risk
Stannard et al., 2012 [27]	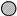 Low Risk	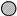 Low Risk	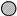 Low Risk	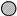 Low Risk	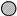 Low Risk	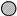 Low Risk
Tauber et al., 2013 [28]	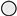 Moderate	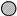 Low Risk	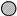 Low Risk	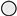 Moderate	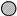 Low Risk	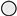 Moderate risk
Vargo et al., 2012 [29]	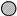 Low Risk	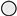 Moderate	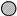 Low Risk	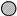 Low Risk	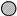 Low Risk	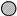 Low Risk
Virani et al., 2016 [30]	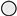 Moderate	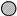 Low Risk	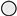 Moderate	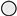 Moderate	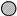 Low Risk	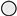 Moderate risk
Svensson-Björk et al., 2021 [31]	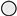 Moderate	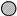 Low Risk	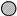 Low Risk	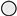 Moderate	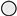 Moderate	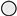 Moderate risk
Svensson-Björk et al., 2022 [32]	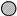 Low Risk	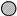 Low Risk	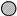 Low Risk	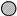 Low Risk	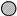 Low Risk	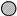 Low Risk

Bias 1: bias arising from the randomization process; Bias 2: bias due to deviations from the intended intervention; Bias 3: bias due to missing outcome data; Bias 4: bias in the measurement of the outcome; Bias 5: bias in the selection of the reported result.

**Table 2:** Findings of the included articles.

Authors and Year	Design and Methodology	Key Results	Conclusions
Anglen et al., 2005 [17]	Experimental study with animal models and human patients (n = 50).	Low-pressure irrigation was more effective in reducing bacterial contamination without tissue damage.	Low-pressure irrigation is an efficient and safe alternative for managing open wounds.
Arti et al., 2016 [18]	Prospective randomized clinical trial with 90 patients treated and followed for one month.	NPWT showed shorter healing times but no significant differences in infection rates.	NPWT accelerates the healing of open wounds and may be more cost-effective than conventional dressings.
Blackham et al., 2013 [19]	Retrospective review of 200 patients treated with various topical agents on open wounds.	The use of topical antiseptics significantly reduced infections and healing times.	Topical agents are effective and improve clinical outcomes in open fractures.
Blum et al., 2012 [20]	Retrospective cohort study in two trauma centers with 229 open tibia fractures.	Negative pressure therapy reduced deep infection rates from 20.6% to 8.4% (*p* = 0.01).	Negative pressure therapy significantly reduces deep infection rates in open tibia fractures.
Lawrentschuk et al., 2002 [21]	Prospective randomized trial with 50 patients undergoing hip surgery.	PTG dressing significantly reduced blisters compared to NAA dressing (8% vs. 64%, *p* = 0.0028).	PTG produces fewer blisters compared to NAA, making it a more suitable option after hip surgery.
Ondari et al., 2016 [22]	Unblinded randomized clinical trial conducted in a Kenyan hospital with 84 patients divided into two groups.	No significant differences in infection rates were found between the two regimens (23% vs. 19%, *p* = 0.699).	Antibiotics for 24 h are adequate prophylaxis against infections in Gustilo II open fractures.
Rezzadeh et al., 2015 [23]	Randomized controlled trial in 120 patients divided into groups with and without growth factor treatment.	The growth factor-treated group showed greater granulation tissue formation and accelerated healing.	Growth factors significantly improve the healing of complex wounds.
Stanirowski et al., 2016 [24]	Prospective randomized study in 120 patients undergoing major surgery.	Pressure dressings significantly reduced healing times and postoperative infections.	Pressure dressings are effective in improving healing in complex postoperative wounds.
Stannard et al., 2006 [25]	Prospective study in 140 patients with traumatic wounds treated with VAC or standard dressings.	VAC reduced the healing time and the need for reoperations.	Vacuum-assisted closure is effective in accelerating healing and reducing complications.
Stannard et al., 2009 [26]	Prospective study in 160 patients with severe trauma treated in referral hospitals.	NPWT reduced complication rates by 25% compared to standard dressings.	NPWT is an effective option for managing severe lower limb trauma wounds.
Stannard et al., 2012 [27]	Systematic review of the recent literature on emerging technologies.	Technologies like NPWT and smart dressings showed promising results in wound healing.	Technological innovations can significantly transform the management of complex wounds.
Tauber et al., 2013 [28]	Retrospective cohort study with 180 patients in two trauma hospitals.	The use of topical antiseptics significantly reduced infection incidence (*p* < 0.05).	Topical antiseptics are effective in reducing infections in open fracture wounds.
Vargo et al., 2012 [29]	Longitudinal study with 5-year follow-up in 250 surgically treated patients.	Functionality was significantly better in patients with early intensive rehabilitation.	Intensive rehabilitation improves functional outcomes in severe lower limb fractures.
Virani et al., 2016 [30]	Multicenter prospective study with 200 patients treated with silver dressings or conventional bandages.	Silver-based dressings significantly reduced infection rates (12% vs. 25%, *p* < 0.05).	Silver-based dressings are effective in reducing infection rates in open fracture wounds.
Svensson-Björk et al., 2021 [31]	Multicenter randomized clinical trial with 377 incisions (uni- and bilateral), comparing negative pressure therapy and standard dressings.	No significant differences in infection incidence were found between the two groups at 90 days postoperatively.	The routine use of negative pressure therapy is not recommended for low-risk incisions after EVAR.
Svensson-Björk et al., 2022 [32]	Cost-effectiveness analysis based on data from the INVIPS randomized clinical trial, considering procedure-related costs and the quality of life.	Negative pressure therapy significantly reduced infection incidence, with an incremental cost of €1.853 per infection avoided.	Negative pressure therapy is a cost-effective strategy for reducing infections in open vascular inguinal surgeries.

NPWT: Negative Pressure Wound Therapy; PTG: polyurethane foam dressing; NAA: non-adherent absorbent dressing; VAC: vacuum-assisted closure; INVIPS: inguinal negative pressure therapy in patients with vascular surgery study.

## Data Availability

Not applicable.
